# 

**Published:** 1888-05

**Authors:** 


### AMERICAN JOURNAL

—OF—

### DENTAL SCIENCE,

—EDITED BY—

F. J. S. GORGAS, M. D., D. D. S.

VOLUME XXII—Third Series.

1888–89.

BALTIMORE:

SNOWDEN & COWMAN,

LONDON:

TRUBNER & CO. 60 PATERNOSTER ROW.

1889.

32
5 / 14
172.
SURGEON GEN'L'S OFFICE
LIBRARY
122790

### Index to Volume XXII.---Third Series.

Annual Commencement of the Baltimore College of Dental Surgery, - - - - - - 526

A Wonderful Germicide, - - - - - - 335

Aluminium in the Arts and Manufactures, - - 381

A Case in Office Practice, - - - - - - 55

An Impromptu Intero Dental Splint, - - - 425

A New Aluminum Process, - - - - - 373

A Doctor's Narrow Escape, - - - - 237

A Case of Epithelioma of the Gums, with a Few Remarks, 80

American Dental Association, - - - - 190

A Few Items of Importance, - - - - - 323

A Review of J. C. Reeve, M. D., on "Anæthetics in Dental Practice," - - - - - - 337

An Incorrect Statement, - - - - - 426

A Reaction Against Antiseptics, - - - - 422

Anæsthetics in Dental Practice, - - - 273

Aluminum, - - - - - - - 249

Amalgam in Contact with Gold, - - - - 191

A Plea for Tube Teeth, - - - - - 459

A Double Tragedy, - - - - - 235

Biting the Finger Nails, - - - - - 383

Bichloride of Mercury in Dental Practice, - - 164

Blind Abscess, - - - - - - 142

Beef, Milk and Phthisis, - - - - - 432

Brom-Ethyl as an Anæsthetic, - - - - 423

BIBLIOGRAPHICAL.

Vick's Floral Guide, - - - - - 475

The Physician's Visiting List, - - - - 474

Pearson's Dentists' Appointment Book, - - 475

Harris' Principles and Practice of Dentistry, - - 574

Cigarette Smoking, - - - - - - 334

Curious Fatality Which Pursues the First Born Sons of the Crockett Family, - - - - - - 327

Correspondence Between F. J. S. Gorgas, Dean of University of Maryland Dental Department, and Dr. T. S. Waters, President of National Association of Dental Examiners, - - - - - - 278

Chicago Dental Society, - - - - - - 522

Causes of Mortality, - - - - - - 336

Cleaning the Teeth, - - - - - - 46
Cleft Palate and Artificial Velum, - - - - - 89
Copper Amalgam, - - - - - - 173
Certain Points Connected With the Structure of Dentine, 349
Cocaine in Surgery, - - - - - - 357
Copper Amalgams, - - - - - 416
Chloroform Administration, - - - - - 561
Chemistry, Physiology and Pathology of Nitrous Oxide—
Practical Points, - - - - - - 515
Do all Amalgams Shrink? - - - - - 332
Detection of Concealed Insanity by the Use of Nitrous
Oxide, - - - - - - 336
Dead and Diseased Teeth and their Treatment, - 269
Death While Under the Influence of Nitrous Oxide Gas, 330
Destroying Pulps, - - - - - - 143
Death by Electricity. The New Method of Executing
Criminals, - - - - - - - 384
Diseases of the Maxillary Sinus or Antrum, - 518
Do Benign Tumors Become Malignant? - - 446
Diseases Outside the Mouth that Affect the Oral Structures, 64
Dental Origin of Pyæmia, - - - - - 139
Deciduous Teeth and the Evils of their Decay and Loss, 71
Dental Hygiene, - - - - - - 451
Dental Treatment of Children, - - - - 542
Dental Inlaying With Porcelain, - - - 562
Devitalizing Pulps, - - - - - 133
Disinfection of Surgical Instruments, - - - 431
Electricity in Dentistry, - - - - - 363
Extirpation of the Larynx, - - - - - 432
Effects of Mental Overwork, - - - - 331
Early Rising and Longevity, - - - - - 478
Erythrophlæine as a Local Anæsthetic, - - 239
Empyema of the Antrum, - - - - - 124
Epistaxis: Lemon Juice, - - - - 240
Former Illiberality in the Profession, - - - 43
Food Cranks, - - - - - - 477
Fifth, Sixth, Seventh and Eighth Districts Dental Societies
of New York State, - - - - - 461
Faith Healing, - - - - - - 501

Galvanoplastic Tooth Crowns, - - - - 84

Georgia State Dental Society, - - - 289

History of Dentistry, - - - - - 493

Hypnotism at Berlin, - - - - 382

Hurry is the Cause, - - - - - 477

How Shall the Surgeon Disinfect His Instruments? 427

Headache from Eye Strain, - - - 481

Immediate Filling of Root Canals, - - - 397

Implantation with Clinical Demonstration, - 49

Immediate Insertion of an Artificial Lower Jaw After

Removal of the Natural One, - - - 558

In favor of a Vegetable Diet, - - - - 48

Joint Meeting of the American Dental Association and the

Southern Dental Association, - - - 252

Labial and Palatine Cavities—Cause, Mode of Preparation, and With What to Fill Them, - - - 223

Leaves From My Experience, - - - 454

Micro-Organisms and the Teeth, - - - 184

Mercury, - - - - - - - 48

Mouth Microbes, - - - - - 97

Mistaken Diagnosis, - - - - 476

Missionary Dentists, - - - - - 480

Medical Men and Dentists, - - - 480

Make Your Work a Pleasure, - - - - 45

OBITUARY.

Dr. George W. Keely, - - - - 238

Dr. Thomas Brian Gunning, - - - 473

Odontological Society, - - - - 31

Oxygen Not so Important in Respiration, - - 431

Opium Smoking, - - - - - 432

Pivot Teeth, Crowns, Etc., - - - - 366

Pyorrhœa Alveolaris, Etiology and Treatment, - 205

Perry's Remarks in Conjunction With Bonwill on Amalgams and Anticipation, etc. - - - - 258

Programme for the Joint Session of the American and

Southern Dental Associations, - - - 135

Professional Patents, - - - - - 145

Peroxide of Hydrogen, - - - - 176

Prejudice Against Amalgam, - - - - 192

Painless Excavation by Anæsthetizing the Tooth with Ether Spray, - - - - - - 131

Pyorrhœa Alveolaris with Practical Cases, - - 23

Prosecution of American Dentists, - - - 232

Resuscitation in Threatened Death from Chloroform, - 129

Reflex Pain Following the Extraction of a Tooth, 479

Some Difficult Cases and their Treatment, - - 168

Some Affections of the Gums. - - - 193

Swaging Machine, - - - - - - 144

Some Suggestions on Metal Cap Crowns. - - 456

Some Practical Points in the Physiology and Pathology of the Fifth Pair of Nerves in Relation to Inveterate Neuralgia - - - - - 59

Special Pathology of the Wisdom Teeth, - - 109

Sweets, - - - - - - - 47

St. Louis Dental Society, - - - - 375

Some Affections of the Gums, - - - 241

South Carolina Dental Association, - - - 180

Septic Root Canals, - - - - - 553

Treatment of Pain, - - - - - - 1

The Combination of Metals for Filling Teeth, - 8

The Tariff on Instruments, Books and Drugs, - 187

The National Association of Dental Examiners, - 190

The Requisite Conditions for Practicing Dentistry in the Province of Quebec, - - - - - 93

The Implantation of Teeth in Artificial Sockets, - 319

Treatment of Proximate Surfaces, - - - - 529

The Sixth Year Molar, - - - - 229

The Glands, - - - - - - 333

Toothmarks Made by a Burglar Lead to His Capture, 329

The Relation of Social Life to Surgical Disease, - 305

The University of Maryland Dental Department and the Secretary of the National Association of Dental Examiners, - - • - - - 375

The International Tooth Crown Company *versus* The Dentists of the United States, - - - 571

The Careful Finishing of Amalgam Fillings, - 556

The Infant-Food Problem, - - - - - 74

The Management of Pulpless Teeth from the Standpoint of Daily Practice, - - - - - 157

The Tonsils, (Faucial, Lingual, Pharyngeal and Discrete); their Functions and Relation to Affections of the Throat and Nose, - - - - - 385

The Spread of Antisepsis, - - - - - 429

The Transplanting of a Rabbit's Cornea into the Human Eye, - - - - - - - 412

The Influence of Maternal Impressions on the Fœtus, 402

The Seventh Annual Commencement of the University of Maryland Dental Department, - - 523

Teeth with Dead Pulps, Without Fistule, and the Filling of Roots, - - - - - - 262

Treatment of Pulpless Teeth, - - - - 433

The Care of the Nails, - - - - 476

Treatment of Burns, - - - - - 240

Treatment of Carbuncle, - - - - 428

The Philosophy of a Cold, - - - - 47

The Successful Transplanting of a Piece of Rabbit's Cornea into the Human Eye, for the Purpose of Restoring Sight to a Blind Man, - - - - - 118

Treating Roots, - - - - - 549

The Hands, - - - - - 439

The Dental Department of Iowa University, - - 568

University of Maryland Dental Department, 327, 472, 424

Ulcerative Diseases of the Mouth, - - - 216

Unhealthy Breath, - - - - - - 16

Virchow on Artificial Deformities, - - - 430

Verdict of Malpractice in Administration of Chloroform, 431

What Cocaine to Use? - - - - 288

Where do Teeth Decay? - - - - 335

Who is My Neighbor? - - - - 478

Waste, - - - - - - 44

What Shall We Eat? - - - - - 576

What Do You Know About Amalgam? - - 511

### *To Readers and Correspondents!*

Communications intended for publication, and exchange journals and papers should be addressed to the EDITOR OF THE AMERICAN JOURNAL OF DENTAL SCIENCE, 845 N. Eutaw St., Baltimore, Md. All letters relating to the business of the JOURNAL, or containing cash remittances, to be directed to Messrs. SNOWDEN & COWMAN. Articles for publication should be received by the editor at least three weeks previous to the first month on which the number in which it is designed for them to appear, is issued.

### CONTENTS:

ARTICLE I.—Treatment of Pain. By James Maughan, M. D ........... 1

" II.—The Combination of Metals for Filling Teeth. By Dr. C. T. Tockwell .......... ........... 8

" III.—Unhealthy Breath. By Alfred H. Tester, L. D. S ........ 16

" IV.—Pyorrhœa Alveolaris with Practical Cases. By J. L. Gish, M. D., D. D. S ...................... 23

" V.—Odontological Society....... ........................ ........ 31

### MONTHLY SUMMARY.

Former Illiberality in the Profession...... .... ........................ ..... 43

Waste........................................ ............ ............ ...... 44

Make Your Work a Pleasure................. ... ................ ........... 45

Cleaning the Teeth ........ ............ ................................ ........ 46

Sweets.......... ........................ ............................................ 47

The Philosophy of a Cold.............................................. 47

In Favor of a Vegetable Diet .. .............. ........................... ...... 48

Mercury ........................... .............................. ........... 48

☞ The AMERICAN JOURNAL OF DENTAL SCIENCE will contain fifty pages of reading matter in each number, making a volume of about *Six Hundred* pages,

EXCLUSIVE OF ADVERTISEMENTS.

